# Social Determinants of Health are Associated with Coping of Informal Caregivers of Adults with Heart Failure

**DOI:** 10.1177/10547738231223790

**Published:** 2024-01-30

**Authors:** Austin Matus, Ryan Quinn, Michael A. Stawnychy, Gladys Thomas, Miatta Goba, Jenna Garo, Deborah Gordon, Barbara Riegel

**Affiliations:** 1 Institute for Diabetes, Obesity and Metabolism, University of Pennsylvania Perelman School of Medicine, Philadelphia, PA, USA; 2 University of Pennsylvania School of Nursing, Philadelphia, PA, USA; 3 Hospital of the University of Pennsylvania, Philadelphia, PA, USA

**Keywords:** social determinants of health, stress, coping, caregivers, caregiver burden, heart failure

## Abstract

We explored the influence of social determinants of health (SDH) risk on stress and coping style in heart failure (HF) caregivers. In this cross-sectional study, data from 250 caregivers were analyzed. Multivariable linear regression analyses were performed to determine the extent to which SDH risk (measured using a modified PRAPARE tool (National Association of Community Health Centers), range 0–22) predicted stress (Perceived Stress Scale, 0–56) and coping style (active (0–45), avoidance (0–30), and minimization (0–30)) while accounting for caregiver burden (HF Caregiver Questionnaire (HF-CQ) 0–100). Multivariable regression analysis with backwards elimination variable selection approach was used to identify which SDH risk factors best predicted coping styles. SDH risk was significantly associated with avoidance and minimization coping styles. Each unit increase in SDH risk was associated with an increase of 0.6 ± 0.2 units (*p* = .0008) in avoidance and 0.7 ± 0.2 units (*p* < .0001) in minimization coping style. Race and “supporting others” significantly predicted avoidance coping style; scores were 3.3 ± 0.8 units greater for caregivers who were not White (*p* < .0001) and 1.4 ± 0.5 units greater (*p* < .01) for each additional person whom they supported. Race significantly predicted minimization coping style; scores were 4.4 ± 0.7 units greater for caregivers who were not White (*p* < .0001). Caregivers with higher SDH risk may avoid and minimize to cope with caregiving challenges.

**Justice, Equity, Diversity and Inclusion**

1. Lay Language Summary of Findings

We explored whether social determinants of health (SDH) risk predicted stress and coping of caregivers of patients with heart failure. SDH risks included factors such as economic stability, education, and social support. We found that caregivers with higher SDH risk reported greater avoidance and minimization to cope with caregiving stress.

2. Justice, Equity, Diversity, and Inclusion (JEDI)

We incorporated the principles of Justice, Equity, Diversity, and Inclusion into the design of the study by addressing systematic barriers to participation in research such as lack of transportation, time, and internet access. Our efforts allowed for the recruitment of a diverse sample of individuals from racial and ethnic minorities and vulnerable populations. Our findings suggest that individuals facing greater SDH risks may be less likely to adopt active coping styles, which are thought to be most effective. Interventions to support active coping in underserved and/or vulnerable populations are needed.

## Introduction

The social, physical, and economic conditions of a society influence health. Evidence suggests that individuals facing socioeconomic inequalities, such as those who are poorer and less educated, have greater health problems and higher rates of premature mortality than those who are wealthier and more educated (Lewer et al., 2020; Raghupathi & Raghupathi, 2020). These differences in health outcomes are not experienced exclusively by those at the extreme ends of society but rather on a gradient by all. The social gradient in health outcomes, in part, results from inequities in the immediate circumstances within which individuals live, such as differences in access to healthcare and education as well as conditions of living, leisure, and work. Thus, the World Health Organization has coined the wider set of forces and systems shaping the conditions in which a person is born, lives, works, and ages as the social determinants of health (SDH) (World Health Organization, 2022). Individuals with higher exposure to SDH risks, such as those experiencing economic instability, inequities in access to quality education and healthcare, unsafe living environments, and social isolation (Office of Disease Prevention and Health Promotion, n.d.), are susceptible to negative health effects of stress.

The demands of informal caregiving are stressful (Fasiku et al., 2022; Grant & Graven, 2018) and these demands may surpass a caregiver’s ability to adapt resulting in a high caregiver burden (Liu et al., 2020; Pinquart & Sörensen, 2003). Caregivers who fail to develop effective coping styles are at risk of becoming care recipients themselves, as ineffective coping may lead to increased self-care neglect (Swartz & Collins, 2019; von Känel et al., 2019), stress, anxiety, and depression, each of which are risk factors for poor health outcomes (AARP and National Alliance for Caregiving, 2020). This is especially true in caregivers with high SDH risks, a group for whom health outcomes are generally poorer (Grady & Rosenbaum, 2015; Marquis et al., 2019). Caregivers in this group may have unstable employment, lower educational attainment, and difficulty accessing high-quality healthcare services. They may live in environments with health and safety risks, such as high violence rates or unsafe water, and lack social support (Office of Disease Prevention and Health Promotion, n.d.). Each of these factors may exacerbate the impact of informal caregiving on their health (Leykum et al., 2022).

**Figure 1.**
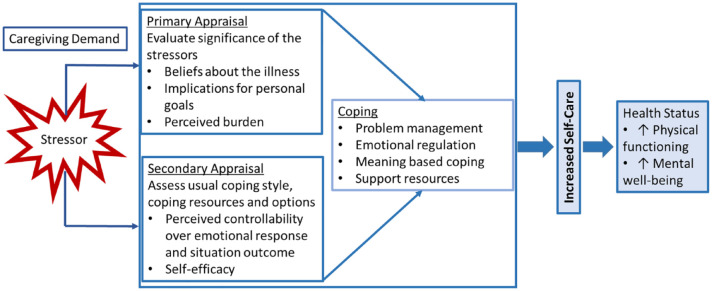
Transactional model of stress and coping. Reprinted from Contemporary Clinical Trials, Vol 85/2019, B. Riegel et al., Health coaching to improve self-care of informal caregivers of adults with chronic heart failure—iCare4Me: Study protocol for a randomized controlled trial, 105845, Copyright (2019), with permission from Elsevier.

The Transactional Model of Stress and Coping (Lazarus & Folkman, 1984) (Figure 1) illustrates how SDH risk may influence health status in informal caregivers. In the model, a stressor like caregiving initiates a dynamic person–environment transaction of primary and secondary appraisal and coping behaviors, or things that people do to avoid being harmed by life strains (Pearlin & Schooler, 1978; Riegel et al., 2019). Primary appraisal entails an assessment of the significance of the stressor, yielding a perception of burden. Informal caregivers of individuals with a chronic illness such as heart failure (HF) are routinely burdened with the support of a loved one who is compromised in caring for themselves.

Regardless of the specific stressor, secondary appraisal involves the assessment of resources available to help cope with the perceived burden. Inequities of the immediate environment for those with high SDH risks, such as lack of transportation to get to a hospital or insufficient funds to afford ambulatory services, render these individuals more likely to feel ill-equipped to control arising issues. This appraisal process informs the coping style. Coping plays a vital role in determining the impact of a stressor, as how people deal with stress can reduce or amplify the short-term and long-term emotional impact of a stressor and physical and mental health (Skinner et al., 2003; Zajdel et al., 2023).

Coping styles may be active, avoidant, or minimizing (Saban et al., 2021; Smyth & Yarandi, 1996). Active coping styles may be understood as problem-focused coping behaviors such as planning, positive reframing, acceptance, and use of instrumental support (Saban et al., 2021). Avoidant coping style involves avoiding or ignoring a stressor or situation, such as self-distraction, denial, behavioral disengagement, and substance use (Livneh, 2019). Minimization coping style refers to downplaying the significance of a stressor or situation (Saban et al., 2021; Smyth & Yarandi, 1996). While active coping style is problem focused, avoidant and minimization coping styles may be understood as emotion focused (Algorani & Gupta, 2023; Kazemi et al., 2021). Problem-focused coping styles are typically employed when one feels capable of doing something constructive about a stressor. Emotion-focused coping styles are employed predominately when one believes that the stressor is something that must simply be endured (Carver et al., 1989; Folkman & Lazarus, 1980). Active coping style is generally considered helpful because it involves direct action toward handling the underlying problem and has been associated with positive outcomes (Algorani & Gupta, 2023; Gustems-Carnicer et al., 2019; Lazarus & Folkman, 1984). By contrast, avoidant coping style is generally thought to be unhelpful as it typically does not address the underlying issue and may result in increased emotional distress (Algorani & Gupta, 2023; Carver et al., 1989; Saban et al., 2021). Minimization coping style may be either helpful if it supports the maintenance of a positive outlook and emotional well-being despite adversity (Kazemi et al., 2021) or unhelpful if employed singularly, failing to address the underlying issue (Saban et al., 2021; Taylor, 1983). Helpful coping styles may be supported by interventions that address stressor appraisal, promote self-efficacy, and focus on developing skills in helpful approaches (Glanz & Schwartz, 2008; Riegel et al., 2019). Unhelpful coping styles can be addressed by intervention if identified and tailored to fit individual needs (Riegel et al., 2019).

While we have reason to believe that SDH risk impacts coping styles in informal caregivers of patients with HF, this has not been demonstrated in the literature. If such a relationship exists, the nature of it is unknown. To progress science toward improving health outcomes among informal caregivers with high SDH risk, we explored the relationships between SDH risk, stress, and coping styles in a sample of adults caring for patients with HF. Then, we sought to identify which elements of SDH risk were the best determinants of coping styles.

## Methods

### Participants

In this cross-sectional study, we analyzed baseline data from 250 caregivers participating in an ongoing randomized controlled trial (RCT) assessing the efficacy of a virtual support intervention to improve self-care of informal caregivers of adults with HF (Riegel et al., 2019). All participants were informal caregivers providing care at least 8 hours/week, who demonstrated poor self-care (as indicated by the Health Self-Care Neglect scale (Riegel et al., 2023) scores ≥2) and were able to complete the study protocol (i.e., adequate vision and hearing, able to read and speak English). Caregivers were excluded from participating if they demonstrated cognitive impairment (Telephone Interview for Cognitive Status score (Brandt et al., 1988; Soldati et al., 2021) <25), were enrolled in another support trial, had an untreated major psychiatric illness (e.g., schizophrenia), reported an inability to use technology, or if the patient for whom they were caring was enrolled in hospice or receiving end-of-life care. The Institutional Review Board approved the study protocols, and all participants provided written informed consent to participate.

### Measures and Calculations

All measures used in this secondary analysis were collected during RCT enrollment. SDH risk was measured with PRAPARE, modified to include binary items for sex, need to support others, and early retirement due to caregiving (Weir et al., 2020). Race was categorized in the measure as White or not White. Three items were removed that were not relevant to our study population (farm worker status), were measured in-depth by another instrument (stress), or were already required to participate in the study (English proficiency). The remaining 14 items assessed individual-level structural determinants (i.e., sex, race, ethnicity, employment, veteran status, education, income, insurance, material security, transportation, social integration, social support, need to support others, early retirement for caregiving). Items were tallied to yield a total score (Range: 0–22). Eleven of the 14 items have a maximum score of one point, two items have a maximum score of two points (i.e., transportation and supporting others), and one item has a maximum score of seven points (i.e., material security). Higher scores indicate greater SDH risk (see Supplemental material 1 for tally scoring breakdown) (Brister, 2021).

Caregiver burden was included as an indicator of the caregiving load experienced by each caregiver and measured with the HF-CQ (Baik et al., 2022; Stromberg et al., 2017). The HF-CQ is a 21-item scale with three domains: physical, emotional/psychological, and lifestyle (range: 0–100). This instrument has demonstrated strong internal consistency reliability and responsiveness to change (Stromberg et al., 2017).

Stress was measured with the Perceived Stress Scale (Cohen et al., 1983, 1994), a 14-item instrument scored on a five-point Likert scale (range: 0–56), where higher scores indicate a greater feeling that life is unpredictable, uncontrollable, and overloaded. Scores from 0 to 18 may be considered low stress, 19 to 36 may be considered moderate stress, and 37 to 56 may be considered high stress (Higgins, 2018). Coping styles were measured using a modified version of the Ways of Coping Scale (Smyth & Yarandi, 1996) containing 35 items scored on a four-point Likert scale comprising three scales: active coping style (range: 0–45), avoidant coping style (range: 0–30), and minimization coping style (range: 0–30). Active coping style is operationalized as aggressive efforts to alter a situation, avoidant coping style as wishful thinking and efforts to escape or avoid problems, and minimization coping style as efforts to detach oneself from an unpleasant situation (Saban et al., 2021; Smyth & Yarandi, 1996). Higher scores indicate more use of that style. In addition, a relative score is computed by dividing each scale’s mean score by the sum of all three scales’ mean scores.

### Analysis

Summary statistics were computed to describe caregivers’ clinical and sociodemographic characteristics using means and standard deviations as well as frequencies and percentages for continuous and categorical measures, respectively. To assess the internal consistency of the HF-CQ instrument, Cronbach’s alpha was computed. To explore the extent to which the SDH risk score is predictive of stress and coping style, separate multivariable linear regression analyses were performed for each outcome: Perceived Stress Scale score, active coping score, minimization coping score, and avoidance coping score. Each model included the SDH risk tally and the HF-CQ score as explanatory variables.

In addition, exploratory multivariable analyses were conducted to assess the extent to which individual SDH risk domains were predictive of stress and coping style. For each outcome that exhibited a significant association with SDH risk in the preliminary analysis, a multivariable model was constructed using a backward elimination variable selection approach. Each of the 14 SDH risk variables was included as predictors. Predictor variables were removed from the model until all remaining variables exhibited a statistically significant effect. The caregiver burden was forced to remain in each model for theoretical reasons, as described above. Analysis was performed using SAS version 9.4 for Windows (SAS Institute Inc.). An alpha level of .05 was used for statistical significance.

## Results

The sample (*n* = 250) was predominately White, female older adults with SDH risk tally scores consistent with the general population (Luzius et al., 2022), moderate perceived stress scores, and high caregiver burden scores compared to previous studies (Thodi et al., 2023). Most caregivers were spouses of the patient, reporting an average caregiving duration of 7 ± 9.5 years and providing care for a median of 3.25 hours per day (Table 1). The use of active coping style was most common (42%), while minimization (33%) and avoidance (25%) coping styles were less prevalent. The HF-CQ instrument exhibited excellent internal consistency (Cronbach’s alpha = .94).

**Table 1.** Participant Characteristics (*n* = 250).

Demographics	
Race (*n* (%))	
Black	74 (29.7%)
White	155 (62.2%)
Asian	4 (1.6%)
Hispanic or Latino	1 (0.04%)
Other	4 (1.6%)
Multi-racial	11 (4.4%)
Sex (*n* (%))	
Female	213 (85.2%)
Age (mean ± SD)	55.3 ± 13.6 years
Relationship with the patient (*n* (%))	
Spouse of patient	149 (59.8%)
Child of patient	34 (13.7%)
Parent of patient	32 (12.9%)
Other	35 (14%)
Time as a caregiver (mean ± SD)	7.0 ± 9.6 years
Hours caregiving/day (median)	3.25 hr
Scale scores (mean ± SD)	
SDH risk tally total (range: 0–22)	4.4 ± 2.3
Race (*n* (%))	1 Point: 86 (34.54%)
Sex (*n* (%))	1 Point: 213 (85.2%)
Ethnicity (*n* (%))	1 Point: 9 (3.6%)
Social integration (*n* (%))	1 Point: 160 (64%)
Education (*n* (%))	1 Point: 51 (20.6%)
Employment (*n* (%))	1 Point: 140 (56.2%)
Income (*n* (%))	1 Point: 37 (15.4%)
Insurance (*n* (%))	1 Point: 100 (40.8%)
Veteran status (*n* (%))	1 Point: 11 (4.47%)
Quit job to provide care (*n* (%))	1 Point: 49 (19.7%)
Social support (*n* (%))	1 Point: 25 (10.0%)
Transportation (range: 0–2; *n* (%))	1 Point: 9 (3.6%) 2 Points: 5 (2.0%)
Supporting others (range: 0–2; *n* (%))	1 Point: 42 (16.9%) 2 Points: 44 (17.7%)
Material security (range: 0–7; *n* (%))	1 Point: 31 (12.4%) 2 Points: 7 (2.8%) 3 Points: 5 (2.0 %) 4 Points: 1 (0.4%) 5 Points: 0 (0.0%) 6 Points: 1 (0.4%) 7 Points: 0 (0.0%)
Caregiver burden (range: 0–100)	38.46 ± 22.9
Perceived stress (range: 0–56)	26.2 ± 7.6
Coping scales	
Active coping [range: 0–45] (relative score)	22.7 ± 9.7 (42% ± 14%)
Avoidant coping [range: 0–30] (relative score)	9.9 ± 6.2 (33% ± 12%)
Minimization coping [range: 0–30] (relative score)	12.3 ± 5.5 (25% ± 11%)

*Note.* SDH = social determinants of health.

When assessing the extent to which SDH risk was predictive of stress and coping styles, the total tally of SDH risk was not a significant determinant of stress (Table 2). Caregiver burden, however, was a significant predictor of stress; for each one unit increase in the HF-CQ burden score, the stress score was estimated to increase by 0.2 ± 0.02 units (*p* < .0001).

**Table 2.** Multivariable Regression Models of Stress and Coping Styles.

Parameter	Estimate	Standard error	*p*-Value
Model of perceived stress (*n* = 245)			
Intercept	17.08	0.97	**<.0001**
HF-CQ total	0.21	0.02	**<.0001**
PRAPARE risk Talley score	0.23	0.17	0.18
Model of active coping (*n* = 243)			
Intercept	22.03	1.63	**<.0001**
HF-CQ total	−0.03	0.03	.32
PRAPARE risk Talley score	0.41	0.30	0.17
Model of minimization coping (*n* = 243)			
Intercept	9.92	0.90	**<.0001**
HF-CQ total	−0.01	0.02	0.42
PRAPARE risk Talley score	0.68	0.17	**<.0001**
Model of avoidance coping (*n* = 241)			
Intercept	4.83	0.96	**<.0001**
HF-CQ total	0.07	0.02	**<.0001**
PRAPARE risk Talley score	0.57	0.17	**<.001**

*Note.* Bold indicates significant findings; HF-CQ = HF Caregiver Questionnaire.

SDH risk was found to significantly predict both minimization coping and avoidance coping styles. Specifically, as SDH risk increased, caregivers’ use of minimization (*b* = 0.68 ± 0.17, *p* < .0001) and avoidance (*b* = 0.57 ± 0.17, *p* < .001) coping styles were estimated to increase. Furthermore, for each one unit increase in the HF-CQ burden score, avoidance coping style was estimated to increase by 0.07 ± 0.01 units (*p* < .0001). Neither SDH risk nor caregiver burden significantly predicted active coping style.

Exploratory multivariable analysis (Table 3) revealed race and “supporting others” to be the SDH risk domains that most saliently predicted avoidance coping style. Participants who were in a racial/ethnic group other than White were estimated to have mean avoidance coping style scores 3.3 ± 0.8 units greater than those of participants who were White (*p* < .0001). For each additional person supported by a caregiver, the mean avoidance coping style score was estimated to increase by 1.4 ± 0.5 units (*p* < .01). Race was also identified as the most salient predictor of minimization coping style; participants who were not White were estimated to have mean minimization coping style scores that were 4.4 ± 0.7 units greater than White participants (*p* < .0001).

**Table 3.** Exploratory Models of Avoidance and Minimization Coping.

Parameter	Estimate	Standard error	*p*-Value
Model of minimization coping (*n* = 242)			
Intercept	14.86	0.78	**<.0001**
Race	4.37	0.71	**<.0001**
HF-CQ total	0.01	0.01	.45
Avoidance coping (*n* = 240)			
Intercept	8.25	0.86	**<.0001**
HF-CQ total	0.08	0.02	**<.0001**
Race	3.30	0.76	**<.0001**
Supporting others	1.41	0.46	**<.01**

*Note.* Bold indicates significant findings; HF-CQ = HF Caregiver Questionnaire.

## Discussion

This study analyzed the relationship between SDH risk, stress, and coping styles among informal caregivers of adults with HF while accounting for caregiver burden. While total SDH risk was not identified as a significant determinant of stress, it was significantly associated with both avoidance and minimization styles. As SDH risk increased, caregivers tended to rely more on avoidance and minimization coping styles. Among the various SDH risk factors contributing to cumulative risk, race other than White and the role of “supporting others” were identified as the primary factors influencing avoidance coping style, while race other than White alone was the primary factor influencing minimization coping style. Although caregiver burden was a significant determinant of stress, it was only associated with avoidance coping styles and no other coping styles. The findings of this study enhance our understanding of SDH risk and caregiving factors that contribute to caregiver stress and coping styles used.

SDH risk demonstrated a significant positive association with minimization and avoidance coping styles among caregivers in our study. Despite this, SDH risk was not associated with stress. Previous literature has demonstrated that adults facing SDH risks such as housing and food insecurity may be more likely to report avoidant coping styles such as emotional eating, using cigarettes, and drinking alcohol when stressed (Joseph et al., 2022). However, the lack of significant association between SDH risk and stress was surprising, as SDH risk factors such as lower income and poor social support have been linked to stress previously (Baum et al., 1999; Braveman et al., 2011; Evans & Fuller-Rowell, 2013; Vicerra, 2022). To our knowledge, no studies have measured SDH risks, caregiver burden, and perceived stress together, nor included both SDH risk and caregiver burden together in a model of stress. As research has identified SDH risk factors including sex (García-Mochón et al., 2019; Swinkels et al., 2019), social support (García-Mochón et al., 2019), financial difficulties (Swinkels et al., 2019), and poor access to social and healthcare services (Del-Pino-Casado et al., 2021) as significant determinants of caregiver burden, it is possible that the influence of SDH risk on stress in our model is being captured by caregiver burden. These results suggest that caregivers with fewer coping resources (e.g., material, financial, social, and professional supports) may feel less empowered to deal with stressors actively, and thus rely on approaches such as minimization or avoidant coping styles. The relationship between SDH risk and coping style was further elucidated via our exploratory analysis to identify which SDH risk factors were most influential in these associations.

Among the individual SDH risk factors, two factors were identified as significant in the observed relationships between SDH risk and coping styles. Race other than White was significantly associated with both avoidance and minimization coping styles, whereas “supporting others” was associated with only avoidance coping styles. This finding regarding race is consistent with prior research demonstrating that Black individuals may be more likely to employ avoidant coping styles in response to stressors (Meints et al., 2016; Morrison & Hopkins, 2019). For example, Africultural coping is understood as a culture-specific coping practice of Black Americans and is characterized by practices such as distraction and avoidance, engagement with spiritual and religious activities, and collective and ritual-centered coping (Blackmon et al., 2016; Morrison & Hopkins, 2019). Such culture-specific coping practices may relate to social and contextual stressors such as racism that may pose situations that people feel incapable of confronting actionably. For example, writers on the topic of race argue that racism is an inescapable and painful reality of daily life in the U.S. (Essed, 1990; Utsey & Ponterotto, 1996) due to systemic racism (Bailey et al., 2021; Banaji et al., 2021). Structural racism may manifest in challenges and stressors for caregivers who are not White.

The observed relationship between supporting more than one individual and avoidance coping style is consistent with prior research, which has demonstrated a relationship between greater caregiver burden and higher endorsement in avoidance coping style (Kazemi et al., 2021; Rahmani et al., 2019). Thus, an individual’s socially constructed race and caregiving responsibilities may predispose them to the adoption of avoidant or minimizing coping styles by presenting unique stressors perceived as an inescapable burden upon primary appraisal, and unresolvable upon secondary appraisal. However, there are likely points within the primary and secondary appraisal process that could be coached to assist these caregivers in managing this stressor (Riegel et al., 2019).

Finally, caregiver burden was identified as a significant determinant of stress and avoidance coping style, as we expected, but was not associated with minimization coping style. The observed associations are consistent with previous literature, which has suggested that caregiver burden is significantly associated with stress (Pinyopornpanish et al., 2021; Yigitalp et al., 2017) and avoidance (Kazemi et al., 2021; Rahmani et al., 2019). However, the absence of a relationship between caregiver burden and minimization coping style in this study was unexpected as a relationship between caregiver burden and “distancing” coping, described as relating to denial, distraction, or detachment (Kazemi et al., 2021), was previously identified. However, this prior study did not account for SDH risk in their analysis. Therefore, SDH risk may completely mediate the relationship between caregiver burden and endorsement of minimization coping style. Further investigation in this area is warranted.

Positioning our findings within Lazarus and Folkman’s Transactional Model of Stress and Coping (1984) offers insight into the relationships identified in this study. In this model, primary appraisal (evaluation of stressor significance including illness beliefs, personal goals, and perceived burden) and secondary appraisal (assessment of usual coping habits, coping resources, and options) inform coping efforts. SDH risks like race and supporting others may influence illness beliefs and goals, perceived controllability of a situation, or resources (such as time) to address a situation. Cultural norms associated with race may influence factors such as usual coping style (e.g., Africultural coping) that inform coping in future situations. Furthermore, assessments such as a perceived lack of time (in the instance of caring for others) may encourage avoidant coping efforts as an individual feels they do not have the resources to cope actively.

### Implications for Practice

These findings expand our understanding of factors contributing to caregivers’ stress and coping styles. Specifically, we have identified SDH risk as a significant determinant of coping style, even when accounting for the significant role of caregiver burden. Clinicians should pay particular attention to caregivers identifying as racial/ethnic groups other than White and those providing care to additional individuals beyond the patient with HF. These caregivers may rely on avoidance or minimization, as active, problem-focused coping styles may not feel like an option. With an assessment of SDH risk, providers may facilitate active coping styles through care coordination, education (e.g., cognitive behavioral therapy (Drake et al., 2020)), community referrals (i.e., Food pantries, affordable housing programs, transportation services), and HF support groups for patients and caregivers. By addressing SDH risk, clinicians can help reduce caregiver burden, facilitate helpful coping styles, and support caregiver health.

### Limitations

This study is limited by its cross-sectional design and sampling from a single geographical location in the northeastern U.S. While not as representative of Black and Latino participants as the city in which our study took place (41% Black)(U.S. Census Bureau, n.d.), our sample is more representative than other research in this field (Graven et al., 2020). Strengths of the study include the large sample size, which provides confidence in the validity of the results.

## Conclusion

Caregivers with higher SDH risk may be avoiding and minimizing to cope with the challenges associated with caregiving. Interventions that build resilience may effectively promote helpful coping styles, reduce burden, and mitigate the harmful effects of SDH risk.

## Supplemental Material

sj-docx-1-cnr-10.1177_10547738231223790 – Supplemental material for Social Determinants of Health are Associated with Coping of Informal Caregivers of Adults with Heart Failure Supplemental material, sj-docx-1-cnr-10.1177_10547738231223790 for Social Determinants of Health are Associated with Coping of Informal Caregivers of Adults with Heart Failure by Austin Matus, Ryan Quinn, Michael A. Stawnychy, Gladys Thomas, Miatta Goba, Jenna Garo, Deborah Gordon and Barbara Riegel in Clinical Nursing Research

sj-docx-2-cnr-10.1177_10547738231223790 – Supplemental material for Social Determinants of Health are Associated with Coping of Informal Caregivers of Adults with Heart Failure Supplemental material, sj-docx-2-cnr-10.1177_10547738231223790 for Social Determinants of Health are Associated with Coping of Informal Caregivers of Adults with Heart Failure by Austin Matus, Ryan Quinn, Michael A. Stawnychy, Gladys Thomas, Miatta Goba, Jenna Garo, Deborah Gordon and Barbara Riegel in Clinical Nursing Research
