# Supplementary material for: Social Determinants of Health are Associated with Coping of Informal Caregivers of Adults with Heart Failure
Source: Clin Nurs Res. 2024 Jan 30;33(5):334–43. doi: 10.1177/10547738231223790 (PMC11188556; doi:10.1177/10547738231223790)
Supplement: sj-docx-1-cnr-10.1177_10547738231223790 – Supplemental material for Social Determinants of Health are Associated with Coping of Informal Caregivers of Adults with Heart Failure [file sj-docx-1-cnr-10.1177_10547738231223790.docx]

| **Table S1. SDH Risk Tally Point Allocation** | | |
| --- | --- | --- |
| **Responses with Points Allocated** | **Points Allocated to Tally** | |
| **Sex** | | |
| Female, Not Specified, or Decline to Answer | 1 | |
| **Ethnicity** |  | |
| Hispanic or Latino | 1 | |
| **Race** | | |
| Black or African American, Asian, American Indian/Alaskan Native, Native Hawaiian or other Pacific Islander, Other, Multiple Races | 1 | |
| **Employment** | | |
| Unemployed or Employed part-time | 1 | |
| **Veteran Status** | | |
| Yes, currently serve in military or have served in the past | 1 | |
| **Education Level** | | |
| Less than high school degree, High school diploma or GED | 1 | |
| **Household income** | | |
| Do not have enough to make ends meet | 1 | |
| **Main Insurance** | |  |
| Government insurance (Medicare and/or Medicaid), Veteran’s Administration health plan, military medical plans, or other government-reimbursed care [e.g. Indian Health Service] | 1 |  |
| **Material Security (1 point for each unmet need in past year)** | |  |
| Food, Clothing, Utilities, Childcare, Medicine or health care, Phone, Other | 1 - 7 |  |
| **Transportation (1 point for each):** | |  |
| Yes, lack of transportation has kept me from:   - medical appointments or from getting my medications, and/or - non-medical meetings, appointments, work, or from getting things that I need | 1 or 2 |  |
| **Social Integration: How often do you see or talk to people that you care about and feel close to?** | |  |
| Less than 6 times a week | 1 |  |
| **Social Support Quality: How would you rate the quality of the support you receive from others?** | |  |
| Poor | 1 |  |
| **Supporting Others (1 point for supporting 1 person, 2 points for more than one person)** | |  |
| Yes or Yes, more than 1 person | 1 or 2 |  |
| **Did you have to quit a job or take early retirement in order to provide care for the person for whom you are giving care or support?** | |  |
| Yes | 1 |  |
