# Supplementary material for: Social Determinants of Health are Associated with Coping of Informal Caregivers of Adults with Heart Failure
Source: Clin Nurs Res. 2024 Jan 30;33(5):334–43. doi: 10.1177/10547738231223790 (PMC11188556; doi:10.1177/10547738231223790)
Supplement: sj-docx-2-cnr-10.1177_10547738231223790 – Supplemental material for Social Determinants of Health are Associated with Coping of Informal Caregivers of Adults with Heart Failure [file sj-docx-2-cnr-10.1177_10547738231223790.docx]

| **Table S2. Participant Instrument Scores by Racial Category** | | | | |
| --- | --- | --- | --- | --- |
| **Scale** | **Black**  *(n = 73)* | **White**  *(n = 155)* | **Other**  *(n = 20)* | **p-value** |
| **Scale Scores** *Mean ± SD* | | | | |
| **SDH Risk Tally Total** (Range: 0-22) | 5.4 ± 2.6 | 3.7 ± 1.6 | 6.3 ± 3.4 | **< .001** |
| **Caregiver Burden** (Range: 0 -100) | 35.6 ± 22.1 | 38.8 ± 21.9 | 43.8 ± 29.9 | .332 |
| **Perceived Stress** (Range: 0-56) | 25.0 ± 7.6 | 26.6 ± 7.7 | 26.8 ± 6.5 | .307 |
| **Coping Scales** | | | | |
| **Active Coping** (Range: 0-45) | 26.9 ± 10.0 | 20.3 ± 9.0 | 27.2 ± 7.8 | **< .001** |
| **Avoidant Coping** (Range: 0-30) | 11.8 ± 6.6 | 8.8 ± 5.6 | 12.1 ± 6.6 | **<.01** |
| **Minimization Coping** (Range: 0-30) | 15.0 ± 6.1 | 10.9 ± 4.6 | 14.8 ± 5.8 | **< .001** |

**Table S2. Participant Instrument Scores by Racial Category.** Data are Mean ± SD; Other, Participants from all racial groups captured excluding those identifying exclusively as Black or White; SDH, social determinants of health.
